# Current developments of *Psychological Research* and the use of AI

**DOI:** 10.1007/s00426-026-02246-0

**Published:** 2026-02-11

**Authors:** Tilo Strobach, Farid Pazhoohi

**Affiliations:** 1 Department of Psychology, MSH Medical School Hamburg, Am Kaiserkai 1, Hamburg, 20457 Germany; 2 University of Plymouth, Plymouth, UK

Writing an editorial for a scientific journal such as *Psychological Research* provides an opportunity to address important issues for the journal and its context. Since the appointment of the new Editor-in-chief (Strobach, 2025) and the associated reorganization of the editorial board (Strobach & Estudillo, 2025), we have worked a lot together to address the issue of the integrity of the articles and findings published in this journal. This focus has been accompanied by the establishment of the article types “Registered Reports” and “Registered Replications” and other measures, such as a priori power analyses, which are mandatory for today’s submissions to *Psychological Research*. These measures aim to increase the integrity of scientific findings among the psychological-experimental research community. However, over the past year, we have also learnt very clearly that this integrity is more necessary than ever to take a self-assured and confident position outside this community as well. We have learnt that this position is also essential in relation to the society and government institutions, which until recently supported psychological and other research initiatives in their independence. However, we see a worldwide trend that these institutions more and more start to marginalize or undermine this independence. The remarkable history of *Psychological Research* with its fate during Germany’s regime in the 1930s and 1940s should serve as a warning to us today (Heuer, 2022), showing how societal, political, and governmental movements can influence supposedly independent science, scientific institutions, and scientific journals in practical terms.

Another movement that is challenging the integrity of science in general and psychological-experimental research in particular is artificial intelligence (AI), beginning in 2023 following public access to ChatGPT in November 2022. AI systems learn statistical regularities from large datasets to generate, classify, or transform information, producing outputs that are fluent and often difficult to distinguish from human-generated content. In research contexts, they can assist with idea development and study design, literature review and synthesis, data handling and analysis, writing and editing, publication support, communication, and ethical compliance (Khalifa & Albadawy, 2024). However, because such generative systems optimize for plausibility rather than truth, they may fabricate references, distort findings, obscure analytic assumptions, and reproduce embedded biases (Lund et al., 2023).

Our aim is to use this editorial to show the journal’s seriousness concerning AI and to maintain the trust of the journal’s readers in our work. To do so, we think that now more than ever, rigorous peer review is important. The more expert the reviewer is on the topic, especially those with published papers on the subject and cited in the submission, the higher the quality of the peer reviews the journal receives (Resnik & Elmore, 2016). In many instances where we have identified papers that challenge scientific integrity (e.g., fabricated papers or plagiarism), the reviewers had papers on that topic and were also cited in the reference list. This perspective on the importance of expert reviewers who are highly active in relevant research fields is consistent with the general strategy of the publisher of this journal. Despite the potential benefit of AI, Springer Nature also stresses that it is more important than ever to have the involvement of a human in all steps of the publication process (https://link.springer.com/brands/springer/journal-policies).

Another risk of AI is that papers do not contain reliable findings. To reduce this risk, we aim to more actively invite and encourage replication studies and apply, among others, *Psychological Research*’s new article type “Registered Replications” (Strobach & Estudillo, 2025), because replications are also more important than ever. In cases where results may be fabricated (something that is often impossible to detect during peer review), the value of replication becomes critical. Emphasizing and favoring well-designed replication studies would strengthen the journal’s as well as science’ integrity. One specific measure of the journal could be to launch and host an entire Special Collection of replication studies beyond regular “Registered Replications”. Therefore, this is an invitation to interested and potential guest editors to actively participate in hosting such a Special Collection.

Another point concerns the journal’s emphasis. Currently, the focus is on firm experimental, sound empirical designs, robustness, and methodological rigor foundations on the one hand. On the other hand, we also aim to publish papers of novelty and impact, answering “big questions”. In the current scientific climate, rigor may be more important than ambitious but fragile claims, while it is still important to focus on the big questions (Nosek et al., 2012). While both aspects, rigorous experimental grounds versus big questions, are sometimes difficult to combine, it should be our priority to have the best balance between these aspects and to answer big questions with high-quality research!

Finally, the publisher (and so our journal) requires no disclosure regarding language editing of their manuscripts any longer (https://link.springer.com/brands/discover/policies). That means that the use of AI tools for copy-editing purposes does not need to be declared. In this context, the publisher defines the term “AI-assisted copy editing” as AI-assisted improvements to human-generated texts for readability and style, ensuring that the text is free of errors in grammar, punctuation, spelling, and tone. These AI-assisted improvements might include wording and formatting changes to texts, but this assistance must not include generative editorial work and content creation. In all cases, there must be a human responsible for the final version of the text and an agreement from all authors that the edits represent their original work. Beyond this perspective on language editing, we are planning on taking more actions towards transparency regarding the use of AI tools in the context of our journal. That is, we are planning a disclosure requirement whereby authors who use AI beyond basic language editing will be asked to document this use explicitly, either within the Methods section or in a dedicated subsection on AI use, and to provide representative examples of such use (e.g., prompts or excerpts of AI interactions) as appendices or supplementary materials. Although AI outputs may vary across instances, including illustrative examples of author–AI interactions would nonetheless enhance transparency and allow readers and reviewers to better understand the nature and scope of AI involvement in the research process.

We believe that this approach ultimately serves the authors’ best interests, as clear and explicit disclosure of AI use helps prevent misunderstandings and reduces the risk of suspicion regarding plagiarism or undisclosed external assistance. By transparently documenting how and where AI tools were used, authors can clearly demonstrate intellectual ownership of their work and distinguish their original contributions from AI-supported processes. Such openness protects authors against potential ethical concerns, supports fair evaluation during peer review, and promotes trust between authors, reviewers, and readers. This way, we are prepared to steer the ship *Psychological Research* into the future and to shape the way our discipline brings the scientific news to the world.

## References

[CR1] Heuer, H. (2022). From Psychologische forschung to psychological research: A rough journey through a century. *Psychological Research Psychologische Forschung*, *86*(8), 2309–2320. 34357423 10.1007/s00426-021-01573-8 PMC9674713

[CR2] Khalifa, M., & Albadawy, M. (2024). Using artificial intelligence in academic writing and research: An essential productivity tool. *Computer Methods and Programs in Biomedicine Update,**5*, Article 100145.

[CR3] Lund, B. D., Wang, T., Mannuru, N. R., Nie, B., Shimray, S., & Wang, Z. (2023). ChatGPT and a new academic reality: Artificial intelligence-written research papers and the ethics of the large language models in scholarly publishing. *Journal of the Association for Information Science and Technology,**74*(5), 570–581.

[CR4] Nosek, B. A., Spies, J. R., & Motyl, M. (2012). Scientific utopia: II. Restructuring incentives and practices to promote truth over publishability. *Perspectives on Psychological Science,**7*(6), 615–631. 26168121 10.1177/1745691612459058 PMC10540222

[CR5] Resnik, D. B., & Elmore, S. A. (2016). Ensuring the quality, fairness, and integrity of journal peer review: A possible role of editors. *Science and Engineering Ethics,**22*(1), 169–188. 25633924 10.1007/s11948-015-9625-5

[CR6] Strobach, T. (2025). Editorial: Start of a new editor-in-chief. *Psychological Research Psychologische Forschung,**89*(1), 45.

[CR7] Strobach, T., & Estudillo, A. (2025). Updates from the journal. *Psychological Research Psychologische Forschung*, *89*(5), 136. 40884578 10.1007/s00426-025-02165-6

